# Effects of a physical education intervention on children’s physical activity and fitness: the PROFIT pilot study

**DOI:** 10.1186/s12887-024-04544-1

**Published:** 2024-01-24

**Authors:** Luiza Naujorks Reis, Cézane Priscila Reuter, Ryan Donald Burns, Clarice Maria de Lucena Martins, Jorge Mota, Adroaldo Cezar Araujo Gaya, João Francisco de Castro Silveira, Anelise Reis Gaya

**Affiliations:** 1 Graduate Program in Human Movement Sciences, Federal University of Rio Grande do Sul (UFRGS), Porto Alegre, RS Brazil; 2 Graduate Program in Health Promotion, University of Santa Cruz do Sul (UNISC), Santa Cruz do Sul, RS Brazil; 3 Department of Health, Kinesiology, and Recreation, University of Utah, Salt Lake City, UT USA; 4 Research Centre of Physical Activity, Health and Leisure, Laboratory for Integrative and Translational Research in Population Health (ITR), University of Porto, Porto, Portugal

**Keywords:** Physical education, Physical exercise, Physical performance, Food and nutrition education

## Abstract

**Background:**

Physical education classes are widely accepted as one of the most effective settings for promoting physical activity and health and have often been used to implement physical activity interventions. The aim of this pilot study was to test a physical education intervention program on physical activity levels and physical fitness in a sample of school-age children.

**Methods:**

Participants were a convenience sample of 50 children (34 experimental group and 16 in the comparative group) aged between 6 and 11 years old (Mean = 8.28 years). A 21-week intervention was implemented, consisting of high-intensity and physical fitness-focused exercises, in addition to a once-a-month extra class nutritional education. The following variables were evaluated before and post-intervention: physical fitness, sedentary behavior (SB), light physical activity (LPA), moderate physical activity (MVA), and vigorous physical activity (VPA). Propensity score analyses calculated the average treatment effect on the treated (ATET) within a quasi-experimental framework.

**Results:**

Physical fitness variables showed improvements after the intervention, specifically for agility (ATET = -0.67 s; *p* < 0.001), cardiorespiratory fitness (ATET = 89.27 m; *p* = 0.045), lower limbs power (ATET = 4.47 centimeters; *p* = 0.025), and speed (ATET = -1.06 s; *p* < 0.001). For physical activity and SB levels, there were no improvements after intervention implementation.

**Conclusion:**

The intervention program showed preliminary effectiveness to improve physical fitness of children, but not SB nor physical activity.

**Supplementary Information:**

The online version contains supplementary material available at 10.1186/s12887-024-04544-1.

## Background

Today’s youth are struggling to meet physical activity recommendations, as demonstrated by low moderate-to-vigorous physical activity (MVPA) engagement [1–3], prolonged times spent in sedentary behaviors [2, 4, 5], and low health- and skill-related physical fitness [6, 7]. There is ample evidence demonstrating that greater amounts of physical activity, especially at higher intensities, yield several health-related benefits (e.g. improved physical fitness, decreased adiposity levels, cardiometabolic, bone, cognition and mental health, among others), whereas a higher time being sedentary is associated with poorer health outcomes (e.g. higher adiposity levels, reduced physical fitness, less favorable cardiometabolic profile, among others) [8]. Concerning health-related physical fitness levels, the literature also has been providing evidence that higher levels are associated with healthier profiles [9, 10].

High-intensity interval-based programs have been investigated as a potent and time-efficient form of increasing MVPA [11] and, consequently, potentially promoting children’s physical fitness [12, 13]. Also, low levels of physical fitness seem to lead youth to be physically inactive [14]. Physical education classes are widely accepted as one of the most effective and strategic environments for promoting physical activity, as they have been often used for the implementation of physical activity interventions because of children’s daily attendance at school, and its infrastructure for physical activity-based actions [15]. However, interventions carried out in the school environment need further investigation. Multinational cross-sectional study demonstrated that attending physical education classes was associated with greater amounts of physical activity and lower level of sedentary behaviors [16]. Additionally, previous studies have demonstrated how interventions with short duration high-intensity interval training for children and adolescents were successfully incorporated during school shifts [17].

A possible effective type of physical activity intervention program that can be applied during physical education classes that integrates neuromuscular training was proposed by Faigenbaum et al. [18]. It consists of general body training activities in addition to strength, conditioning, agility, and endurance activities, core stability exercises, and plyometric exercises [18, 19]. In fact, the promotion of children’s physical activity should focus not only on improving a specific component of physical fitness, but on both strength, skill, and aerobic activities [14]. Likewise, current guidelines recommend a combination of aerobic and strengthening activities to promote overall health [8, 20]. Lastly, accumulated evidence has suggested that physical education interventions should be focused on planned and structured quality-based classes, once it seems that duration alone is not enough for improving children’s physical fitness and health components [21, 22]. Also, a healthy lifestyle is multifactorial, and an approach addressing various multidisciplinary components (e.g., physical activity, physical fitness, and a healthy diet) can be effective in promoting it [23].

A common benefit from physical education intervention programs for children is the improvement of cardiorespiratory fitness [24]. Also, intervention programs seem to consistently increase children’s MVPA during physical education lessons in addition to their physical fitness [11, 25]. However, whether there is a substantial increase in the amount of time of physical activity at higher intensities children accumulate during the whole day within their leisure time in addition to school-time is not clear [26]. Also, there are gaps in the literature in terms of ideal intervention execution procedures, primarily with regard to its outcome on promoting children health. The hypothesis of the present study is that after the intervention there will be a decrease in sedentary time and an increase of higher intensity physical activities because of the improvements of physical fitness. Therefore, this study aimed to pilot test a quality-based physical education intervention program on physical activity levels accumulated during the whole day and physical fitness amongst school-age children.

## Methods

### Study design

This pilot study was conducted within a quasi-experimental study framework utilizing non-randomized comparative and experimental groups defined by school affiliation. The study is part of the “Sport Health at School Project” developed for a convenience sample of elementary children aged 6 to 11 years of age, from the 1st to the 5th grade of two public schools in the city of Porto Alegre, southern Brazil.

### Participants

Two schools were invited to participate in the program. The selection of schools occurred due to a pre-existing agreement with the University, based on the mandatory internships in the Physical Education undergraduate curriculum. Additionally, at that time, state schools did not have physical education classes in the curriculum up to the 5th grade of elementary school, taught by qualified Physical Education teachers. A random draw was held to define which schools would make up each group, being organized as follows. School 1: experimental group (EG), received the special program of physical education (PROFIT; which is a circuit training aimed at improving physical fitness and fundamental motor skills) and nutritional education. School 2: comparative group (CG), continued receiving physical education classes with the regular teacher.

**Fig. 1:**
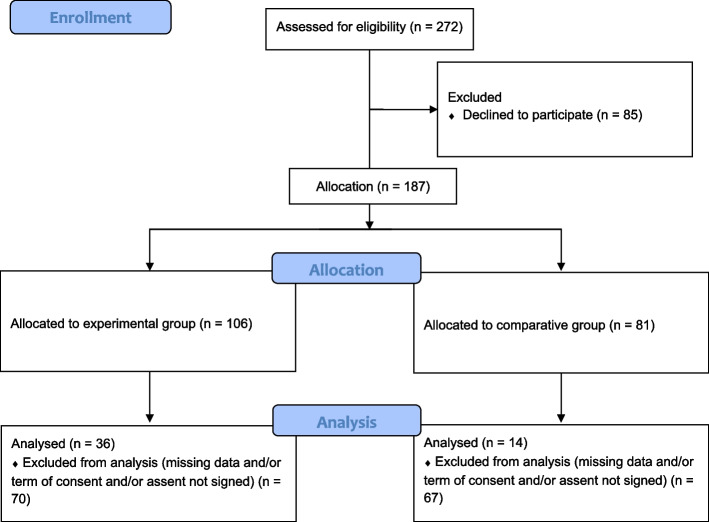
Sampling process

 After acceptance of the participating schools, the researchers went to the school, talked to the students, and sent a note to their parents inviting them to a meeting. At the meeting, the study’s objectives, and procedures were explained to the parents. Although all children from 1st to 5th grade who wanted to participate could attend the program classes, only those whose informed consent was signed by their parents participated in the sample, evaluations, and final analysis, according to the following inclusion criteria: (I) the parent / guardian and the child have signed the informed consent and assent forms, respectively; (II) no contraindications for blood collection; (III) not currently participating in any other physical exercise program; and exclusion criteria: (I) incapacity for physical exercise; (II) use of medications that might interfere with the results; (III) more than three consecutive absences from the intervention program classes. At the end of the assessments, all children received an individual report with their data and explanations concerning the health implications of each indicator. The final sample consisted of 50 children (36 in the EG and 14 in the CG) who completed all assessments. A visual representation of the sampling process can be seen in Fig. 1.

### Experimental group

Physical education session plan and intervention schedule are available in the Supplementary materials 1 and 2. Briefly, the program was planned and conducted by a group of trained researchers who were qualified physical education teachers and nutritionists. Sessions were applied twice a week during physical education lessons, lasting 60 min each throughout the school year (19 weeks). The physical education sessions were organized into 5 stages (with PROFIT being the main differential): 10 min of commuting to the sport court and warm-up; 15 min of circuit training (PROFIT); 10 min of gymnastics components and rhythmic activities; 15 min of motor skills and pre-sports games; and 5 min of rest activities and feedback.

The warm-up consisted of aerobic recreational activities. The PROFIT circuit was based on the studies [19] and the proposal of Faigenbaum et al. [18], and consisted of four stations with exercises aiming at developing the components of physical fitness. The circuit was performed twice with each participant staying at each station for one minute, with an interval of 30 s between them. For progression, the load and complexity of the exercises were changed according to age group. Before the intervention, the cardiorespiratory fitness test was performed and, according to the observed performance, the participants were divided into four groups with similar conditioning characteristics to compose the stations. During classes, the participants should reach at least 75% of their maximum heart rate (indicating moderate-to-vigorous intensity), which was controlled via one participant from each group wearing a portable heart rate monitor (Polar Team2 Pro, Polar, Finland). Participants were assigned to groups of approximately five people with similar performance in the cardiorespiratory fitness test, and each week a different participant would use the heart rate monitor. Part of the components of gymnastics and rhythmic activities was carried out for the development of motor skills. The exercises consisted of throws, jumps, kicks, etc. Finally, the pre-sports games consisted of handball, volleyball, Frisbee, wrestling, among others.

Additionally to the physical activity intervention, a nutritional intervention was developed by a nutritionist and consisted of actions carried out once a month for participants and their parents. The activities were held at the school, for approximately 45 min and consisted of challenges and nutritional information transmitted playfully. For parents, materials were sent on the themes worked with the children and nutritional education content [27]. A description of the schedule and actions is provided in the Supplementary material 3.

### Comparative group

Comparative students continued with physical education classes taught by regular teachers with content not structurally planned with the aim of improving physical fitness. Control of content and classes by researchers was not applied.

### Assessments

Assessments were carried out before and after the intervention implementation. Initial assessments were carried out by trained researchers over 2 months for both groups in their respective schools. Anthropometric and physical fitness assessments took place during the initial two weeks. For the use of the accelerometer, meetings were held with parents or guardians, and explanatory notes were sent to those who could not attend. The accelerometers were placed and removed from the children in the schools themselves, upon prior appointment with their parents or guardians. In addition, parents were sent an explanatory note on how to proceed with the device. The same procedures were followed in the post-intervention evaluations.

### Anthropometrics

The anthropometric assessment was carried out with the children barefoot and wearing light clothing. Height was measured on a metal stadiometer (Filizola) with a resolution of 1 mm and body mass on an analog-digital scale (Filizola) with a resolution of 0.1 kg. The body mass index was calculated using the following formula: body mass (kg)/height² (m).

### Physical fitness

Abdominal muscular endurance, agility, cardiorespiratory fitness, flexibility, lower limbs power, speed, and upper limbs power were evaluated using the procedures suggested by PROESP-BR. This is a battery of tests designed to assess health parameters and physical performance at a very low cost, using minimal sophisticated materials. It is easily accessible and applicable for physical education classes, while rigorously adhering to criteria of validity, reliability, and objectivity. These criteria can be checked in the online website and in the manual [28].

Abdominal muscular endurance was evaluated using the 1-minute sit-up test. When testing the number of sit-ups in 1 min, the child should do the maximum number of sit-ups in one minute. To do this, position yourself in the supine position, with your knees bent at 45º and your arms crossed over your chest. While the evaluator holds the children’s ankles, they move the trunk to flexion, touching the elbows to the thighs and returning to the starting position.

Agility was evaluated using the square test. A square measuring 4 m on a side was measured, with a cone arranged at each angle. The children moved as fast as possible, diagonally, then to the left (or right), going to the other diagonal and ending up towards the initial cone, always touching the cones with their hands. The children had two chances and the best moment was noted in seconds with two decimal scales.

Cardiorespiratory fitness was evaluated using the 6-minute running and walking test, the participants were divided into small groups. They were advised on the importance of running as much as possible, keeping a constant pace, and avoiding walking and running peaks. During the test, the passage of time at 3 and 5 min was reported. At the end of the test, the students remained in the place where they were, so that the distance covered during the 6 min (in meters) could be recorded (number of laps multiplied by the size of the court, added to the meters in the last return).

Flexibility was evaluated using the sit-and-reach test. The barefoot children, with their knees extended and hands overlapping, should leaned slowly, extending their hands forward as far as possible, remaining in the position as long as necessary for the distance to be noted. Two attempts were made, the best result in centimeters was recorded.

Lower limbs power was evaluated using the horizontal jump test, a measuring tape was fixed to the floor, perpendicular to the starting line. The children were instructed to remain standing with their feet parallel, behind this mark. They performed the jump as far as possible with both feet simultaneously. Each child had two attempts and the greatest distance was recorded in centimeters.

Speed was evaluated using the 20-meter run test. Children moved as quickly as possible, passing through three parallel lines previously marked: a start line, one 20 m from the first (timeline), and another 21 m from the exit (finish line). The third line served as a reference for the child not to slow down before crossing the 20 m. Time was recorded in seconds with two decimal scales.

Upper limbs power was evaluated using the medicine ball throw test. A measuring tape was fixed to the floor perpendicularly to the wall, with the zero points of the measuring tape fixed to the wall. The children sat with their backs flat against the wall, legs together and knees extended. Then they flexed their arms and threw the ball as far as possible, keeping their backs against the wall. The distance was recorded in centimeters from point zero to the place where the ball hit the ground for the first time in two attempts, and the best one was noted.

#### Physical activity and sedentary behavior

The physical activity levels were assessed using Actigraph accelerometers (wActiSleep-BT Monitor). The equipment was placed on the children’s waists using an elastic strap, in the midaxillary line on the right side. Children were instructed to use it for seven consecutive days, including weekdays and two weekend days, throughout the day, only removing it for water activities. The minimum amount of accelerometer data that was considered acceptable for analysis purposes was four days (including at least one weekend day), with at least 10 h/day of usage time, after removal at sleep time. Data were analyzed using Actilife software (ActiGraph®, version 5.6, USA) and collected at a sampling rate of 30 Hz, downloaded in one-second periods, and aggregated for 15-second periods. For counting the counts for cutoff points of accelerometers, the ready cutoff of Evenson et al. [29] was used for periods of 15 s (≤ 25 counts/15 seconds for sedentary behavior, between 101 and 573 counts/15 seconds for light physical activity, and ≥ 574 counts/15 seconds for moderate to vigorous physical activity).

### Statistical analysis

A descriptive analysis was performed to describe the subjects before and after intervention implementation using means and standard deviations (SD) or absolute and relative frequencies. The difference in differences (D.i.D.) analysis was performed. Change scores for each dependent variable were calculated (Δ = Posttest minus pretest scores). The bootstrapping resampling procedure for the Analysis of Covariance (ANCOVA) adjusted for sex, age, and the outcome variable at pretest was used to verify differences between experimental and comparative groups. All bootstrapping procedures used a resampling of 1,000 samples and the Bias-Corrected and Accelerated (BCa) method. The effect sizes (Cohen’s *d*) for the ANCOVAs were computed. The values of *d* < 0.49 indicated a small effect size; 0.50 < *d* < 0.79 indicated a medium effect size; and *d* > 0.80 indicated a large effect size [30]. Cohen’s *d* values were also used as a measure of standardized difference for the Student *t*-tests between experimental and comparative groups at pretest. These analyses were carried out using the Statistical Package for the Social Sciences (SPSS, v. 23.0 IBM, Armonk, NY) software. Post hoc power analyses were conducted for the ANCOVAs in G*Power (v. 3.1.9.7) software to verify if there was at least 80% statistical power in the models. The power computation used Cohen’s *f* as the measure of effect, which was estimated using the following equation: $f= \sqrt{\frac{{R}^{2}}{1-{R}^{2}}}$ (where *R* ^2^ stands for the coefficient of determination [30]); in addition to 4 numerator degrees of freedom, 2 groups, and 4 covariates.

Propensity score matching was employed to estimate the average treatment effect on the treated (ATET) for each physical activity level and each physical fitness outcome variable. Propensity score matching may be used in non-randomized quasi-experimental research designs to improve internal validity. Propensity score matching estimators impute the missing potential outcome for each participant by using an average of the outcomes of similar (matched) participants that receive the comparative condition. Similarity (matching) between participants is based on estimated treatment probabilities, which are known as propensity scores, or the predicted probabilities of being within the EG (the treatment) given a set of covariates. In the current study, a logit model was used to predict propensity scores using age, sex, BMI, and the outcome variable at pretest as covariates with matching based on the single nearest neighbor approach. ATET was computed by taking the average of the difference between the observed and potential outcomes for participants within the experimental (treatment) group. Alpha level was set at *p* < 0.05 for all analyses with propensity score matching carried out using Stata v17.0 (StatCorp., College Station, Texas, USA).

## Results

Table 1 presents the descriptive data. There were no differences between experimental and comparative groups for age, abdominal muscular endurance, agility, BMI, flexibility, lower limbs power, and upper limbs power variables before the intervention implementation. However, the CG had better cardiorespiratory fitness (*p* = 0.046; *d* = 0.63; medium difference) and speed (*p* = 0.003; *d* = 0.89; large difference) performance at pretest. Also, there was a lower proportion of females in the CG compared to the EG (*p* = 0.028). The Supplementary Table 1 presents differences at pretest for those who were followed-up and those who were not followed-up in the EG. Participants who where not followed-up exhibited higher BMI scores, but this difference was classified as small (*p* = 0.003; *d* = 0.46).

**Table 1:** Descriptive characteristics

	**Experimental group**	**Comparative group**	**Total**	***p***
	**Mean (SD)**			
Age (Pretest, years)	8.33 (1.33)	8.14 (1.35)	8.28 (1.33)	0.653
Physical fitness (Pretest)				
Abdominal muscular endurance (rep.min^−1^)	25.06 (9.96)	23.31 (8.89)	24.68 (9.45)	0.563
Agility (s)	7.64 (0.93)	7.81 (0.52)	7.70 (0.82)	0.422
BMI (kg.m^−2^)	17.77 (4.23)	18.86 (3.22)	17.85 (4.03)	0.336
Cardiorespiratory fitness (m)	670.17 (150.84)	760.31 (118.24)	700.12 (148.76)	**0.046**
Flexibility (cm)	36.49 (8.10)	41.46 (7.89)	37.52 (8.85)	0.080
Lower limbs power (cm)	109.66 (20.19)	117.00 (17.09)	111.45 (19.35)	0.214
Speed (s)	4.87 (0.59)	4.39 (0.36)	4.73 (0.57)	**0.003**
Upper limbs power (cm)	184.23 (52.07)	177.85 (28.52)	180.20 (48.89)	0.593
Physical activity levels (Pretest; min.day^−1^.week^−1^)				
Sedentary behavior	432.60 (48.99)	414.16 (71.16)	426.70 (56.94)	0.384
Light physical activity	275.62 (55.12)	408.35 (79.31)	318.09 (88.78)	**0.001**
Moderate physical activity	44.03 (12.52)	37.85 (21.31)	42.06 (15.90)	0.298
Vigorous physical activity	21.29 (9.70)	10.53 (7.85)	17.85 (10.39)	**0.001**
Moderate-to-vigorous physical activity	65.33 (20.35)	48.39 (28.06)	59.91 (24.16)	**0.046**
	**n (%)**			
Sex				
Male	15 (41.7)	11 (78.6)	26 (52.0)	0.028
Females	21 (58.3)	3 (21.4)	24 (48.0)	

Data are expressed as mean and standard deviation (SD) or absolute and relative frequencies; Differences between intervention and comparative groups calculated using the bootstrapping resampling procedure for the Student *t*-test for continuous variables; The Fisher’s exact test was used to verify if there were more participants than expected within an specific cell of the contingency table for categorical variables association (*p* < 0.05)

Table 2 presents the D.i.D. analysis for physical fitness and physical activity levels between experimental and comparative groups. After intervention implementation, the EG exhibited improved agility (ΔMean = 0.32 s; *p* = 0.041; *d* = 0.43; small effect size), cardiorespiratory fitness (ΔMean = 113.37 m; *p* = 0.030; *d* = 0.96; large effect size) and speed performance (ΔMean = 1.07 s; *p* = 0.003; *d* = 1.47; large effect size) when compared to the CG. No statistically significant differences were observed for other physical fitness and physical activity variables.

**Table 2:** Difference in differences analysis between experimental and comparative groups

	**Experimental group**	**Comparative group**	**Total**	**Cohen’s ** ***d***	***R*** ^**2**^	**Power**
	**Mean (SD)**					
**Physical fitness**						
Δ AME (rep.min^−1^)	3.69 (7.95)	6.00 (6.00)	4.34 (7.47)	0.31	0.240	0.87
Δ Agility (s)	**-0.69 (0.65)**	**-0.37 (0.95)**	**-0.60 (0.75)**	0.43	0.396	0.99
Δ BMI (kg.m^−2^)	0.17 (1.50)	-0.53 (2.40)	-0.03 (1.80)	0.39	0.167	0.66
Δ CRF (m)	**95.94 (118.32)**	**-17.43 (118.64)**	**63.55 (128.08)**	0.96	0.399	0.99^a^
Δ Flexibility (cm)	0.58 (4.77)	-3.43 (6.85)	-0.54 (5.66)	0.74	0.227	0.84
Δ LLP (cm)	8.78 (18.05)	1.31 (15.85)	6.80 (17.65)	0.43	0.309	0.96^a^
Δ Speed (s)	**-0.49 (0.74)**	**0.58 (0.69)**	**-0.19 (0.87)**	1.47	0.612	0.99
Δ ULP (cm)	21.87 (31.20)	27.54 (28.72)	23.37 (30.37)	0.19	0.476	0.99^a^
**Physical activity levels (min.day** ^**−1**^ **.week** ^**−1**^ **)**						
Δ SB	-13.09 (65.55)	-31.99 (87.64)	-18.88 (72.60)	0.26	0.199	0.76^a^
Δ LPA	151.09 (58.98)	26.07 (110.32)	112.82 (96.59)	1.60	0.555	0.99^a^
Δ MPA	-6.25 (15.82)	5.59 (15.55)	-2.63 (16.52)	0.75	0.269	0.91^a^
Δ VPA	-9.60 (9.81)	-1.90 (6.31)	-7.25 (9.52)	0.86	0.615	0.99^a^
Δ MVPA	-15.88 (23.04)	3.72 (17.63)	-9.88 (23.21)	0.91	0.379	0.99^a^

Data are expressed as mean and standard deviation (SD)

Δ denotes changes in the dependent variable (Posttest minus pretest scores)

Bold denotes the difference between experimental and comparative groups calculated using the Bootstrapping resampling procedure for the ANCOVA adjusted for sex, age, and outcome variable at pretest (*p* < 0.05)

*AME* Abdominal muscular endurance, *BMI* Body mass index, *CRF* Cardiorespiratory fitness, *LLP* Lower limbs power, *ULP* Upper limbs power, *SB* Sedentary behavior, *LPA* Light physical activity, *MPA* Moderate physical activity, *VPA* Vigorous physical activity, *MVPA* Moderate-to-vigorous physical activity

^a^Power calculated using 49 participants

Results from propensity score matching for the physical fitness variables are communicated in Table 3. The ATET was − 0.67 s (95% CI: -1.01 s to -0.33 s; *p* < 0.001) for agility, 89.27 m (95% CI: 1.85 to 176.68 m; *p* = 0.045) for cardiorespiratory fitness, 4.47 cm (95% CI: 1.25 to 18.75 cm; *p* = 0.025) for lower limbs power, and − 1.06 s (95% CI: -1.25 s to -0.86 s; *p* < 0.001) for speed, indicating significant improvements in these fitness variables after intervention implementation. No statistically significant ATETs were observed for abdominal muscular endurance (*p* = 0.109), BMI (*p* = 0.279), flexibility (*p* = 0.351), or upper limbs power (*p* = 0.108).

**Table 3:** Propensity score analysis

	**ATET**	**95% CI**	***p***
**Physical fitness**			
Abdominal muscular endurance (rep.min^−1^)	-3.14	-6.98; 0.70	0.109
Agility (s)	**-0.67**	**-1.01; -0.33**	** < 0.001**
BMI (kg.m^−2^)	0.54	-0.44; 1.52	0.279
Cardiorespiratory fitness (m)	**89.27**	**1.85; 176.68**	**0.045**
Flexibility (cm)	2.34	-2.58; 7.26	0.351
Lower limbs power (cm)	**4.47**	**1.25; 18.75**	**0.025**
Speed (s)	**-1.06**	**-1.25; -0.86**	** < 0.001**
Upper limbs power (cm)	-10.69	-23.73; 2.34	0.108
**Physical activity levels (min.day** ^**−1**^ **.week** ^**−1**^ **)**			
Sedentary behavior	**68.13**	**12.45; 123.81**	**0.016**
Light physical activity	31.57	-35.66; 98.80	0.357
Moderate physical activity	-1.39	-16.78; 13.98	0.859
Vigorous physical activity	1.30	-5.24; 7.83	0.698
Moderate-to-vigorous physical activity	-10.54	-25.50; 4.41	0.167

*ATET* Average treatment effect on the treated; matches for school, age, sex, BMI at pretest, and the outcome variable at pretest

Results from propensity score matching for the physical activity levels variables are also communicated in Table 3. The ATET was 68.13 min.day^−1^.week^−1^ (95% CI: 12.45 min.day^−1^.week^−1^ to 123.81 min.day^−1^.week^−1^; *p* = 0.016) for SB, indicating a significant increase after intervention implementation. No statistically significant ATETs were observed for LPA (*p* = 0.357), MPA (*p* = 0.859), VPA (*p* = 0.698), or MVPA (*p* = 0.167).

## Discussion

The main purpose of this pilot study was to verify preliminary effectiveness of a quality-based physical education intervention program on physical activity levels accumulated during the whole day and physical fitness amongst school-age children. The results suggested significant improvements in agility, cardiorespiratory fitness, lower limbs power, and speed in the participants of the EG after intervention implementation.

The benefits of physical education intervention programs on children’s physical fitness are well documented [24]. Regarding possible confounding variables, such as age, sexual maturation, and weight status, evidence suggest that different youth populations does not benefit themselves equally from school-based intervention. Boys and younger participants, especially those with higher levels of physical activity and lower physical fitness before intervention tend to benefit the most [31]. For this reason, it is worthwhile to note that there were more girls in the EG than the CG, which may explain the lack of improvements for some components of physical fitness and physical activity levels and explain the better performance of some physical fitness components in the CG at pretest. Additionally, there exists the potential for the regression to the mean phenomenon [32], given that the EG exhibited lower cardiorespiratory fitness and speed performances at pretest. However, these pretest scores were added as covariates to control for their effects in the propensity score matching approach.

Reviews have been summarizing insights of the effects of higher intensity physical activity intervention programs on cardiorespiratory fitness in children aged 6 to 12 years over the years [13, 33, 34]. Moreover, there is a common and increasing scientific interest in the promotion of cardiorespiratory and muscular fitness improvements via physical education classes [13, 14], probably because of their importance as independent markers of overall health from childhood/youth to adulthood [35–38]. The intervention implementation within the present pilot study focused on providing a variety of strength and conditioning exercises at higher intensities for the EG, which could explain why physical fitness improvements were observed within the present study since exercise intensity is positively related to fitness and other health outcomes in youth, such as cardiometabolic health and bone mineral density [18, 21, 34]. In addition to cardiorespiratory fitness, there were improvements in agility, lower limbs power, and speed within the EG. The lack of improvements on other muscular fitness components (i.e. abdominal muscular endurance and upper limbs power) may be attributed to the focus on higher aerobic intensity physical activities instead of more resistance-based exercises, agreeing with how the intervention is designed may be a crucial factor for success [39] since usually muscular fitness improvements are observed within physical activity-based [40] and school-based [41] interventions amongst children and adolescents. Moreover, García-Hermoso et al. [22] systematically reviewed the literature and demonstrated that physical education interventions based on quality over quantity may be sufficient to increase health-related physical fitness components because it integrates a conscientiously structured and planned physical education class. The findings of the present pilot study agree with these previous reports, bringing relevance as an adequate public health strategy that can be used to better develop health- and skill-related physical fitness [42].

Concerning the physical activity intensities (sedentary behavior, LPA, MPA, VPA, and MVPA), we hypothesized that after the implementation of our intervention there would be a decrease in sedentary time and an increase of higher intensity physical activities because of the improvements of physical fitness. However, our hypothesis was not reached, since the results indicated no significant increases in physical activity intensities and a significant improvement in SB after intervention implementation. In fact, school-based physical activity programs seem to have a minimal impact on increasing time engagement in MVPA levels. Also, according to Eddolls et al. [17], school-age children seem to compensate for the increase in physical activity levels, with a reduction in the physical activity performed the following day. This hypothesis is called the “*activitystat*” and suggests that increased levels of physical activity during one part of the day or period may result in a compensatory decrease in physical activity elsewhere. This compensatory mechanism may explain why sedentary behavior increased after the intervention. Physical education intervention programs seem to consistently increase children’s MVPA during physical education classes, but they are less consistent on the effectiveness of improving physical activity engagement outside of school during leisure time [11, 26, 34]. From this perspective, probably our results could suggest a future research purpose considering the analyses of physical activity intensities separated by daily context.

Further, physical activity level changes involve not only children individually. Khawaja et al. [43] indicated that school, home, and neighborhood environments can significantly influence the opportunities a child has to engage more in MVPAs. Schools play a pivotal role in health-promoting interventions [15]. However, the school environment is not the only place where MVPA should be encouraged. Home and neighborhood environments, such as parks, playgrounds, and other green areas, in addition to parental permission and support, seem to play a substantial role in the total amount of physical activity children can achieve and possibly increase their amounts of MVPA [44]. There is a consensus indicating that families’ environments are important for supporting and promoting health behaviors [45]. For these reasons, school-based interventions should specifically consider the family, teachers, and peers as important sources of social support for the promotion of general physical activity [46] since multicomponent interventions that involve the entire school environment seem to be the most effective [34].

Despite this short-term intervention did not show improvements in physical activity intensities, it is plausible that the improvements in physical fitness found in the present pilot study could benefit long-term physically activity. Improving physical fitness and maintaining it may be a key component in this strategy because physically fit children tend to maintain healthier levels of physical fitness as they grow-up, whereas ‘weak’ children tend to become ‘weak’ adults [47]. Also, probably these children will not face problems to be active and will be more prone to pursue an active lifestyle and participate more in diversified physical activity that inherently demands healthier levels of physical fitness [48, 49]. Thus, these children will probably improve even more not only agility, cardiorespiratory fitness, lower limbs power, and speed, but their fundamental motor skills and other physical fitness components as well (e.g. muscular fitness and adiposity levels) and probably improve their engagement in physical activities because of future ongoing physical activity participation, as suggested by a conceptual model by Stodden et al. [50]. Moreover, the more engagement in diversified physical activities since early childhood, the more benefits conferred to physical activity at any intensity and thereby a healthy life course perspective [51]. Finally, MVPA engagement seems to naturally decrease as children grow up [52]. Therefore, physical education classes and schools should be kept as fundamental places to provide opportunities of being physically active and maintain physical fitness levels.

From the perspective of the afore-mentioned discussion, future research should target possible confounders beyond the school setting. Additionally, it should explore the long-term implications of physical fitness improvements on physical activity levels and fitness itself in addition to considering physical activity separated by physical education school-time and outside school-time. The present pilot study has some worthwhile strengths. A major strength was the social role of an intervention implementation intended to cause an effect on physical activity and fitness levels. Second, the use of gold-standard device-measures (accelerometry) to assess sedentary behavior and physical activity levels. Also, the use of the propensity score matching approach to correct the fact that there were no random assignments before experimental manipulation and to be able to suggest causal effects. However, randomized controlled trials are still encouraged to properly test if similar intervention designs to the present pilot study cause improvements on physical activity levels and physical fitness. Also, there are a few limitations that should be considered. First, non-randomization, small sample size, and drop-out from pre- to post-test make generalization challenging. The sleep time, the third measure of the 24-Hour Movement Behaviors in addition to sedentary behavior and physical activity, was not considered in the present pilot study. Future studies should focus on all components of the 24-Hour Movement Behaviors, which is also associated with healthier indicators in children and adolescents [53]. The 6-minute running and walking test and the BMI are not direct measures of cardiorespiratory fitness and adiposity, respectively. Gold-standard protocols, such as VO_2peak_ maximum protocol and DXA, should be utilized in future research. This could explain why we did not find improvements in adiposity levels, which is a common finding within school-based intervention programs [54]. We hypothesized that implementing a nutrition intervention in addition to the exercise could maximize physical activity intensities engagement and fitness by reducing weight because multidisciplinary approaches are often required to cause a modification of lifestyle [55]. However, we did not observe an improvement by using only BMI status, which may be attribute to a lack of statistical power. This study did not track long-term levels of physical fitness and physical activity levels within the EG. Lastly, it did not include potential covariates, such as eating habits, income, and neighborhood physical activity opportunities, in this analysis.

## Conclusion

This pilot intervention program showed preliminary effectiveness to improve levels of physical fitness (agility, cardiorespiratory fitness, lower limbs power, and speed), but not for the physical activity intensities. Improving patterns of movement behaviors requires a change in lifestyle habits. Therefore, as the afore discussion, it needs different planning and organization for the intervention implementation with the most effective participation of parents/guardians, teachers, and school.

### Supplementary Information

**Additional file 1:** **Supplementary material 1. **Physical education sessions plan. **Supplementary material 2. **Intervention design schedule. **Supplementary material 3. **Nutrition intervention schedule and activities. **Supplementary Table 1.** Differences between followed-up and not followed-up in the experimental group.

## Data Availability

The database used and analyzed in the present study is not publicly available as its information may compromise the participants’ privacy and consent involved in the research. However, the data are available from the corresponding author (EA), upon reasonable request.

## Abbreviations

ATET: Average treatment on the treated
BMI: Body mass index
CG: Comparative group
D.i.D: Difference in difference
EG: Experimental group
LPA: Light physical activity
MPA: Moderate physical activity
MVPA: Moderate-to-vigorous physical activity
PROESP-BR: Projeto Esporte Brasil
PROFIT: Program of physical education
VPA: Vigorous physical activity
