# Supplementary material for: Effects of a physical education intervention on children’s physical activity and fitness: the PROFIT pilot study
Source: BMC Pediatr. 2024 Jan 24;24:78. doi: 10.1186/s12887-024-04544-1 (PMC10807217; doi:10.1186/s12887-024-04544-1)
Supplement: Supplementary file 1 — Additional file 1: Supplementary material 1. Physical education sessions plan. Supplementary material 2. Intervention design schedule. Supplementary material 3. Nutrition intervention schedule and activities. Supplementary Table 1. Differences between followed-up and not followed-up in the experimental group. [file 12887_2024_4544_MOESM1_ESM.docx]

**Supplementary material 1.** Physical education sessions plan.

| **Session plan** | | | **Time** |
| --- | --- | --- | --- |
| **Initial part** | **Commute to the sports court** | **-** | 5 minutes |
|  | **Warm-up** | Aerobic recreational activities | 5 minutes |
|  | **PROFIT** | Four stations:  1 – Lower limbs power exercises  2 – Speed and cardiorespiratory fitness exercises  3 – Upper limbs power exercises  4 – Agility and cardiorespiratory fitness exercises | 15 minutes |
|  | **Break** |  | 5 minutes |
| **Main part** | **Gymnastic, rhythmic, motor- and skill-related exercises, and/or sport games** | Motor- and skill-related exercises (throws, jumps, kicks, etc) and/or sport games (handball, volleyball, frisbee, wrestling, etc) | 25 minutes |
| **Final part** | **Cool-down** | Assessment and feedback with emphasis on behavioral and attitudinal aspects | 5 minutes |

**Supplementary material 2.** Intervention design schedule.

| **JUNE** | **JULY** | **AUGUST** | **SEPTEMBER** | **OCTOBER** | **NOVEMBER/DECEMBER** |
| --- | --- | --- | --- | --- | --- |
| **FIVE MONTHS OF INTERVENTION** (19 weeks) | | | | | **ASSESSMENT AFTER FINAL REUNION** |
| 3 weeks | 2 weeks | 5 weeks | 4 weeks | 5 weeks | 4/5 weeks |
| PREPARATION | BASE | | BUILD | PEAK |  |
| Education of movements (posture);  Simple movements;  Discipline and getting to know the teachers. | Intermediate exercise  Intermediate movement  Winter break (2 additional weeks)  Note: Increase load / complexity before break | Intermediate exercise  Intermediate movement  Transition to complex movement  Progress to moderate/vigorous intensity | Complex exercise  Complex movement  Moderate-to-vigorous intensity | Complex exercise  Complex movement  Vigorous intensity | - |

* PREPARATION: To adapt the body to training; BASE: To establish strength, speed, agility, and endurance; BUILD: To increase intensity and its limits; PEAK: To consolidate conditioning.

**Supplementary material 3.** Nutrition intervention schedule and activities.

|  | **Actions** | **Description** |
| --- | --- | --- |
| **APRIL** | **Movie** | Presentation of an educational video or animated cartoon introducing concepts and information about nutrition, food, and eating habits. Subsequently, a reinforcement activity was conducted where children cut, drew, and colored images and drawings that recalled the concepts presented in the film. |
| **MAY** | **Food box** | A box containing various foods, including fruits, vegetables, and processed foods such as snacks, filled biscuits, and candies, was brought into the classroom. The activity involved students identifying the food items solely through touch. After revealing the food by taking it out of the box, an explanation was given about the nutritional values of the respective product and its recommended frequency of consumption. Following the completion of the activity, the children cut, drew, and colored images and drawings that reinforced the concepts presented during the activity. |
| **JUNE** | **Sugar content of foods** | Various foods, particularly processed ones commonly consumed by students, were brought into the classroom. The presentation included separately showing the amount of sugar present in each package and portion. Subsequently, a panel was created displaying the products and the sugar content in each food item. |
| **JULY** | **Energy day** | In collaboration with physical education teachers, a day was designated each week for all students to bring fruits as their school snack, preferably on one of the physical education class days. The objective was for students to understand that fruits are excellent sources of energy and to encourage fruit consumption as a snack. |
| **AUGUST** | **Saturday at school** | In conjunction with other participants of PROESP-BR, a Saturday of activities for parents and students was organized. The event included playful activities, sports, workshops, as well as the exhibition of nutritional education projects completed in the classroom. Nutritionists were also present for discussions and guidance on children's nutrition and diet. |
| **SEMESTERLY** | **Facebook page** | A Facebook page was created on the social network where updates and guidance on nutrition were published. This included photos, videos, and information about the activities planned for the students during the semester. This not only kept parents informed but also established a stronger connection between the nutritional education project and parents or guardians. Additionally, it served as an easily accessible communication channel for nutritionists and the school community. |

**Supplementary table 1.** Differences between followed-up and not followed-up in the experimental group.

|  | **Followed-up** | **Not followed-up** | ***p*** |
| --- | --- | --- | --- |
|  | **Mean (SD)** | |  |
| Physical fitness (Pretest) |  |  |  |
| Abdominal muscular endurance (rep.min^-1^) | 25.06 (9.96) | 22.26 (9.75) | 0.072 |
| Agility (s) | 7.64 (0.93) | 7.73 (0.86) | 0.532 |
| BMI (kg.m^-2^) | 17.77 (4.23) | 19.50 (3.25) | 0.003 |
| Cardiorespiratory fitness (m) | 670.17 (150.84) | 701.84 (136.12) | 0.085 |
| Flexibility (cm) | 36.49 (8.10) | 36.92 (8.04) | 0.753 |
| Lower limbs power (cm) | 109.66 (20.19) | 111.15 (26.05) | 0.729 |
| Speed (s) | 4.87 (0.59) | 4.79 (0.71) | 0.486 |
| Upper limbs power (cm) | 184.23 (52.07) | 198.18 (66.75) | 0.214 |
| Physical activity levels (Pretest; min.day^-1^.week^-1^) | |  |  |
| Sedentary behavior | 432.60 (48.99) | 433.62 (46.10) | 0.890 |
| Light physical activity | 275.62 (55.12) | 289.60 (55.57) | 0.106 |
| Moderate physical activity | 44.03 (12.52) | 40.71 (16.59) | 0.208 |
| Vigorous physical activity | 21.29 (9.70) | 20.14 (13.12) | 0.583 |
| Moderate-to-vigorous physical activity | 65.33 (20.35) | 59.81 (27.88) | 0.242 |

*Note*: Data are expressed as mean and standard deviation (SD); Differences between followed-up and no followed-up calculated using the bootstrapping resampling procedure for the one sample *t*-test (*p* < 0.05).
